# Cross Talk between Cancer and Mesenchymal Stem Cells through Extracellular Vesicles Carrying Nucleic Acids

**DOI:** 10.3389/fonc.2016.00125

**Published:** 2016-05-23

**Authors:** Tatiana Lopatina, Chiara Gai, Maria Chiara Deregibus, Sharad Kholia, Giovanni Camussi

**Affiliations:** ^1^Department of Medical Sciences, Molecular Biotechnology Center, University of Torino, Torino, Italy

**Keywords:** exosomes, extracellular vesicles, non-coding RNA, microRNA, long non-coding RNA, tumor stem cells, mesenchymal stem cells

## Abstract

Extracellular vesicles (EVs) are considered to be a novel complex mechanism of cell communication within the tumor microenvironment. EVs may act as vehicles for transcription factors and nucleic acids inducing epigenetic changes in recipient cells. Since tumor EVs may be present in patient biological fluids, it is important to investigate their function and molecular mechanisms of action. It has been shown that tumor cells release EVs, which are capable of regulating cell apoptosis, proliferation, invasion, and epithelial–mesenchymal transition, as well as to suppress activity of immune cells, to enhance angiogenesis, and to prepare a favorable microenvironment for metastasis. On the other hand, EVs derived from stromal cells, such as mesenchymal stem cells (MSCs), may influence the phenotype of tumor cells through reciprocal cross talk greatly influenced by the transcription factors and nucleic acids they carry. In particular, non-coding RNAs (ncRNAs), including microRNAs and long ncRNAs, have recently been identified as the main candidates for the phenotypic changes induced in the recipient cells by EVs. ncRNAs, which are important regulators of mRNA and protein expression, can function either as tumor suppressors or as oncogenes, depending on their targets. Herein, we have attempted to revise actual evidence reported in the literature on the role of EVs in tumor biology with particular regard to the cross talk of ncRNAs between cancer cells and MSCs.

## Introduction

Extracellular vesicles (EVs) have recently been identified to be instrumental in intercellular communication through the exchange of biologically active molecules, in particular, non-coding RNAs (ncRNA) that can not only modulate gene expression locally but also systemically (1–3). EV molecular composition (nucleic acids and protein) is regulated by cell growth conditions, signal molecules, growth factors, etc. (4–6). Apart from healthy cells, tumor cells also release excessive amounts of tumoral extracellular vesicles (T-EVs) found to be rich in specific sets of ncRNAs different from normal cells, which circulate in different biological fluids in the body (7). The role of ncRNAs in the regulation of gene expression has been extensively studied, and various classes of ncRNAs, with different targets and functions, have been identified (8, 9).

Non-coding RNAs are usually divided into two major groups according to their length. These include small ncRNAs (below 200 nt) defined as microRNAs (miRNAs), and the long ncRNAs (lncRNAs; above 200 nt). miRNAs mostly act on RNA by silencing or post-transcriptionally regulating gene expression (10, 11), whereas lncRNA participate in imprinting and gene dosage regulation, using diverse molecular mechanisms. These include complement annealing with genome DNA, scaffolding histone-modifying complexes by acting as either a “sponge” for proteins and miRNAs, and/or as molecular guides within ribonucleoprotein complexes (12). Other important roles played by both ncRNAs include stem cell pluripotency, embryonic development, cell differentiation, and tumorigenesis.

Recent evidence demonstrates dramatic changes that happen in the level and pattern of RNA carried within circulating EVs during tumor development. Depending on their biological properties and content, EVs have been involved in cancer initiation, progression, and pre-metastatic niche formation (13). It has also become evident that EVs may transfer not only functional ncRNA but also DNA, thus modifying gene expression in recipient cells (14–16).

Tumors contain a heterogeneous population of cells, including mesenchymal stem cells (MSCs), endothelial cells, cancer-associated fibroblasts, immune inflammatory cells, and also cancer stem cells (CSCs). Communication between these cells and cells present in the normal surrounding tissue helps tumor-initiating cells to survive, proliferate, invade, and establish metastasis (17–19). This communication is performed not only by cytokines, hormones, and proteins, but also by transcription factors, ncRNAs, and DNA carried by EVs.

In this review, we mainly focus on the role of ncRNAs carried by T-EVs and by MSC-derived EVs (MSC-EVs) in modification of tumor microenvironment with particular regard to the interaction between cancer cells and MSCs.

## Selected ncRNAs Detected Frequently in EVs During Cancer Development

**Figure 1:**
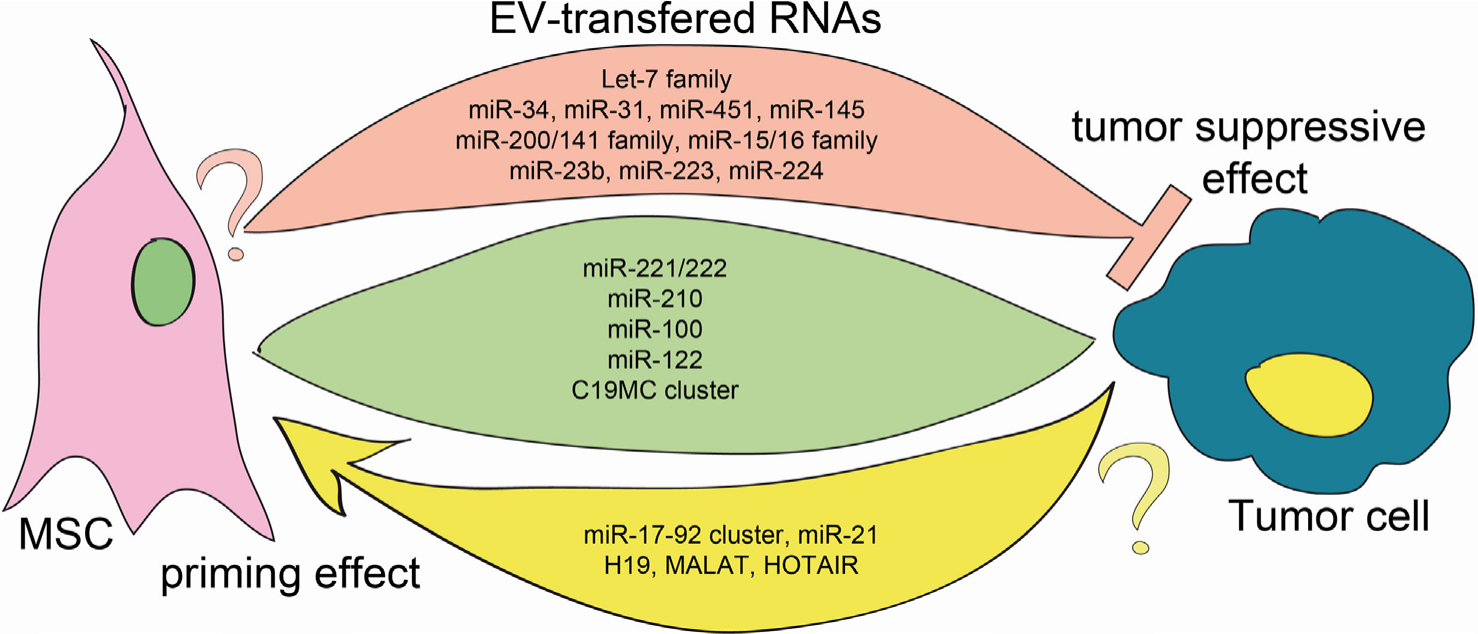
**EV transfer of ncRNA between MSCs and tumor cells**. Green, rose, and yellow flows represent the EV-mediated exchange of information between cells. All mentioned RNAs have been detected in both MSC-EVs and T-EVs. Green flow: RNAs that transferred function in both MSC and tumor cells. Rose flow: it depicts bidirectional transfer of tumor-suppressor miRNAs. Whereas the anti-tumoral action of these EV-carried miRNAs is well established, no data are available on their function on MSC (question mark) (5, 21–28). Yellow flow: it indicates traffic of oncomiRs and oncogenes, that were shown to prime MSCs toward a protumoral phenotype. The action of EVs carrying these RNAs has not been investigated in tumor cells (question mark) (6, 29–32).

The discovery of defined pattern of ncRNA expression in cancer patients has exposed the potential to exploit them as novel diagnostic markers as well as possible therapeutic targets. For instance, Lawrie and colleagues in 2007 reported the presence of miRNAs in the blood of cancer patients and demonstrated their potential as cancer biomarkers (20). Since then, the list of tumor-associated circulating miRNAs has grown evidently and the molecular function of miRNAs in the context of cancer widely elucidated. Interestingly, many of these ncRNAs were also detected within EVs derived from MSCs, therefore possibly indicating a bidirectional function in the communication between tumor cells and stem cells (see Figure 1). However, the functions of different miRNA are not univocal and depending on the context they may exert pro- or anti-tumorigenic activity. Here, we discuss the functions of some miRNAs, miRNA families (i.e., miRNAs sharing similar seed sequence), or clusters (i.e., miRNAs processed from one transcript), which have been reported to be present in both MSC-EVs and T-EVs.

## Let-7 Family

Let-7 family consists of 13 different members, including let-7a-1, 7a-2, 7a-3, 7b, 7c, 7d, 7e, 7f-1, 7f-2, 7g, 7i, mir-98, and mir-202. Furthermore, the presence of these miRNAs has been detected in the circulation of patients with breast, prostate, colon, gastric, oral, and ovarian cancers (21). This cluster of miRNAs are considered to have tumor-suppressor properties as they not only repress genes responsible for self-renewal and “stem” characteristics in cells but also promote differentiation during normal development. Many oncogenes, such as RAS, MYC, HMGA2, and LIN28, are known to be direct targets of let-7 (33). Furthermore, low levels of let-7 have been identified to correlate with more aggressive tumors as well as CSCs (29, 34, 35). Whether let-7 deregulation is the cause of cancer initiation and development or *vice versa* is still unknown.

Extracellular release of let-7 in plasma of cancer patients or in conditioned medium of tumor cells and MSCs has been reported in several studies (36–38). There are two hypotheses about the function of extracellular let-7 in tumorigenesis: the first hypothesis proposes that the release of let-7 through EVs results in the depletion of cells of this miRNA consequently leading to the maintenance of oncogenesis and invasiveness of donor cell. The second theory considers extracellular let-7 as pro-oncogenic based on evidence that it targets caspase-3 (39) and BAX mRNAs (40). In the latter theory, EVs containing let-7 may cause resistance to apoptosis, thus favoring the acquisition of a malignant phenotype by recipient cells.

## miR-21

miR-21 is one of the most frequently upregulated miRNA identified in various different cancer types, including lung, ovarian, breast, colon, gastric, and pancreatic cancers (30, 41, 42). miR-21 is highly enriched in EVs and is considered to be an oncogene, as it promotes tumor cell proliferation, migration, and invasiveness by targeting a number of tumor-suppressor genes, such as various components of the p53 network (43), PTEN (44), and antagonists of the RAS pathway PDCD4, BTG2, SPRY2, and others (45–47). Furthermore, miR-21 also exhibits angiogenic properties as EV-mediated transfer of miR-21 from tumor cells to recipient cells alters their phenotype and promote angiogenesis by enhancing the expression of VEGF (48–50).

Due to its upregulation in cancers and oncogenic properties, miR-21 levels in the plasma has been described as a marker for several types of tumors, such as breast, colorectal, prostate, gastric, ovarian cancer, B-cell lymphoma, glioblastoma, pancreatic cancer, and non-small cell lung cancer (51). Interestingly, this miRNA has also been detected in EVs derived from several stem cells, including MSCs (31). Since MSCs are recruited within the tumor, they may contribute to tumor angiogenesis by releasing miR-21containing EVs.

## miR-17-92 Cluster

The cluster consists of seven different miRNAs: miR-17 (miR-17-5p and miR-17-3p), miR-18a, miR-19a, miR-19b, miR-20a, and miR-92a. Overexpression of this cluster of miRNAs has been observed in several types of cancers (52–56). Numerous studies have shown the presence of these miRNAs in EVs present in plasma or in conditioned medium from cancer cell cultures (42, 51, 57, 58). The molecular mechanisms of pro-tumorigenic action of the miR-17-92 cluster include the targeting of E2F transcription factor family – a critical regulator of cell cycle and apoptosis (59); cyclin-dependent kinase inhibitor CDKN1A (p21) – a potent negative regulator of the G1-S checkpoint (60); and BCL2L11/BIM pro-apoptotic gene (61). Interestingly, it was reported recently that EVs from non-tumor astrocytes were rich in miR-19a and could promote metastatic transformation of recipient tumor cells by targeting PTEN (62). Furthermore, this cluster of miRNAs has also been identified to play a role in tumor angiogenesis by directly targeting anti-angiogenic factors, such as thrombospondin-1 and connective tissue growth factor (63), as well as several other pro-angiogenic proteins, including the integrin subunit alpha5 (64).

miR-92a is an example of miRNA with a dual role in angiogenesis depending on the cell of origin. For instance, when carried by T-EVs, it has been shown to be pro-angiogenic (58), whereas when carried by MSC-EVs it exhibits anti-angiogenic properties (57). This observation suggests that the function of a single miRNA should be considered within a more complex context depending on the interaction with multiple factors.

## miR-15-16 Family

This family includes miR-15a/16-1 cluster (on chromosome 13q14), the miR-15b/16-2 cluster (on chromosome 3q25), and the miR-195/497 cluster (on chromosome 17p13).

The role of miR-15a/16-1 in cancer was suggested by the observation that these genes were down-regulated in B-cell chronic lymphocytic leukemia (65). These two miRNAs function as tumor suppressors, targeting Bcl2, MCL1, and Cyclin D1 genes (66). 15b/16-2 miRNAs are highly similar with miR-15a/16-1 cluster (miR-16-1 and miR-16-2 are identical), but their biological function is controversial, as this cluster has been reported to behave as either a tumor suppressor (67, 68) or oncogenic (69–71).

Expression of miR-15-16 cluster within plasma EVs has been shown by several research groups (51, 72). EVs containing miR-15a and miR-16 are released by different types of vascular cells (such as endothelial progenitor cells, vascular smooth muscle cells, and pericytes) as well as by MSCs. These miRNAs display an anti-angiogenic activity by targeting VEGF-A and AKT3 (73, 74).

## miRNA-200 Family

The miRNA-200 family consists of five highly homologous members: miR-200a, miR-200b, miR-200c, miR-429, and miR-141. They are described as tumor-suppressor miRNAs, which are dysregulated in several malignancies (75, 76). For instance, miR-200c and miR-141 are strong epithelial differentiation inducers of undifferentiated cancer cells (77). They also tend to increase E-cadherin expression and, therefore, cell adhesion, as well as reduce cancer cell migration and invasion (78). Targets of this family of miRNAs include E2F3, E-cadherin suppressor targets, such as Zeb1, Zeb2, and SNAI2, which are important inducers of epithelial–mesenchymal transition and of subsequent tumor cell invasion (79). Additionally, these miRNAs repress Suz12 and Bmi1 stem cell markers whose expression leads to the formation of CSCs (80). Interestingly, other studies suggest a pro-tumorigenic activity of this family of miRNAs, since miR-141 is upregulated in the plasma during prostate (81) or ovarian cancer development (82) and miR-200b has been shown to stimulate epithelial–mesenchymal transition in human tongue cancer cells (70).

## miR-122

miR-122 acts as a tumor suppressor by targeting genes involved in cell proliferation, migration, differentiation, apoptosis, and angiogenesis particularly in hepatocellular carcinoma (83). For instance, it directly down-regulates cyclin G1 that negatively regulates p53 protein stability (84). Other pro-tumorigenic targets of miR-122 include disintegrin, metalloprotease 10 (ADAM10), serum response factor (SRF), and insulin-like growth factor-1 receptor (IGF-1R) (85, 86).

On the other hand, high levels of circulating miR-122 has been shown in hepatocellular carcinoma (87) and, therefore, it is considered as a diagnostic marker for prediction of metastatic progression in other malignancies, including breast cancer (88). Furthermore, T-EVs carrying miR-122 downregulate the consumption of glucose in recipient cells by targeting pyruvate kinase PKM2 and Glucose transporter 1, leading to increased glucose availability for cancer cells. In addition, the same authors showed that T-EV secreted miR-122 also stimulated metastasis (89). It can, therefore, be suggested that miR-122 potentially plays a role during the early stages of tumor growth prior to angiogenesis, when the availability of nutrients is limited, and also when disseminated tumor cells reach distant tissue where they compete with surrounding normal cells for nutrients (89). Therefore, miR-122 has potentially a dual role in cancer development acting as inhibitor and or as an inducer of metastasis depending on the stage of tumor development.

## miR-221/222

miR-221/222 have been characterized as both oncogenic and/or as a tumor suppressor, according to the type of cancer (90). These miRNAs target the oncogene KIT, thus inhibiting tumor growth (91). However, they also target important tumor suppressors, such as PTEN, p27, p57, and TIMP3 (92), and overexpression of miR-221 has been observed in CSCs and also during EMT (93). Furthermore, secretion of miR-221/222 has been shown in the plasma of patients with oral, colon, lung, and other cancers (51). Interestingly, EVs released by MSCs also contain miR-221 and have been implicated to exhibit anti-angiogenic effects (94).

## C19MC Cluster

The miRNAs of C19MC cluster (“chromosome 19 microRNA cluster”) constitutes the largest human miRNA cluster known as of yet (95) as it encodes more than 50 mature miRNAs sharing common seed sequences (96). C19MC is exclusively expressed in the placenta and in undifferentiated cells, being the predominant miRNA species detected in placenta-derived EVs (97). Although the placenta is a normal tissue, it shares several common features with tumors, such as high cell proliferation, lack of cell-contact inhibition, and migratory and invasive properties. Both cancer and placental cells create a microenvironment supportive of immunologic privilege and angiogenesis (98, 99). Some C19MC-miRNAs were classified as oncomiRs as they were associated with invasion and metastasis (100, 101). One such example is miR-520 that was described to play a supportive role in proliferation and invasion due to its ability to suppress CD44 (102) and activate Ras/Raf/MEK/Erk signaling pathways (103). By contrast, this miRNA has also been reported to be involved in suppressing metastasis as it is also attributed toward the direct downregulation of TGFBR2 (104). Nevertheless, amplification of C19MC cluster is considered as a marker characteristic of pediatric brain tumors (100) and the emission of the related oncomiRs has been recently described (105). In addition, it has been shown that C19MC-carring EVs can also exert a possible antiviral effect (106, 107). The pattern of expression of C19MC-miRNAs in embryonic and tumor tissues suggests that exosomes carrying C19MC-miRNAs may play an important role in immunomodulation, cell reprograming, invasion, and angiogenesis.

## Long-Non-Coding RNA

Recently, lncRNAs have been identified to be involved in various human cancers. Many of them have been detected within EVs present in biological fluids. Metastasis-associated lung adenocarcinoma transcript 1 (MALAT1) and HOX transcript antisense RNA (HOTAIR) are the most studied lncRNAs, which are deregulated in majority of cancers (108). They promote tumor growth by regulating cell cycle, invasiveness, migration, epithelial–mesenchymal transition, and tumor angiogenesis.

Metastasis-associated lung adenocarcinoma transcript 1 regulates alternative splicing of pre-mRNAs (109), and influences the expression of different metastasis-associated transcripts (110). HOTAIR downregulates various genes, including HOXA5 – differentiating factor involved in lung development (111); p21 – a mediator of p53-induced growth arrest and apoptosis (112); Wnt inhibitory factor 1 (WIF-1) – an inhibitor of the Wnt/β-catenin pathway that mediates EMT (113); as well as tumor suppressor PTEN – an inhibitor of EMT (114).

In some cases, lncRNAs carried by EVs could enhance its own expression by an autoregulatory loop, as it has been observed with highly upregulated in liver cancer (HULC) lncRNA (115). HULC inhibits expression of tumor suppressor gene p18 and promotes hepatoma cell proliferation. In turn, the inhibition of tumor suppressor gene p18 activity enhances expression of HULC (116).

The biological activity of lncRNAs carried by EVs has been described in CSCs studies. lncRNA H19 carried by T-EVs released from liver CSCs has been shown to promote angiogenesis and endothelial cell pro-adhesive functions (117). H19, as well as other lncRNAs, can not only function as a miRNA sponge for let-7 (118) but also as a precursor for miRNAs, or as an epigenetic modulator (119). Furthermore, H19 has been shown to play a role in tumor angiogenesis, upregulating VEGF and also stimulating heterotypic adhesion between endothelial cells and CSCs (117). Interestingly, the H19 lncRNA has been recently identified in EVs released from MSCs (6).

## DNA Carried by EVs

Extracellular DNA is predominantly found within apoptotic bodies; however, it is also present within microvesicles and exosomes (120, 121) particularly more common in EVs released by tumor cells. Similar to other genetic cargo, EV DNA may also be incorporated and reused by recipient cells (122). Holmgren et al., for instance, reported that DNA secretion within EVs by tumor apoptotic cells occurred independently from nuclear condensation and apoptosis, and that only specific fractions of DNA fragments were present in apoptotic bodies. These fragments were enriched in oncogenes and had the ability to induce malignancy (123). Furthermore, DNA enriched in T-EVs was identified to reflect the genetic status of the tumor, for example, the amplification status of the oncogene c-Myc, as well as retrotransposons such as LINE-1 and Alu (124). This, therefore, means that T-EVs could transfer DNA between cells and insert it into recipient genome using a retrotransposon mechanism (124). It has also been shown that on transfecting non-tumorigenic immortalized rat intestinal epithelial cells (IEC-18) with human Ras oncogene increased the production of EVs rich in double-stranded DNA fragments spanning the entire host genome, including full-length human Ras and other rat oncogenes. In addition, exposure of non-transformed RAT-1 cells to EVs containing mutant H-ras DNA led to enhanced cell proliferation (125), therefore, implying that cancer cells could use EVs for the transfer of DNA oncogenes. Recently, mitochondrial DNA has also been reported to be present in plasma EVs that can be imported into the mitochondria of recipient cells with physiological or pathological consequences (126). Transferred DNA can, therefore, act as a template for both DNA and RNA synthesis (127).

## Cancer Stem Cells Regulate Tumor Environment through EVs: Establishment of Tumor Niche, Metastasis, and Immunomodulation

T-EVs have a number of specific characteristics relevant to tumor development. The acidic microenvironment present around tumors stimulates the release of T-EVs with higher cell fusion capacity and elevated content of the tumor marker Caveolin-1 (128). A number of studies have shown that T-EVs facilitate the escape of tumor cells from the immune system (129). For instance, it has been show that T-EVs carry ligands for death receptors FasL and TRAIL that may induce apoptosis in lymphocytes (130, 131). Furthermore, these EVs could regulate monocyte differentiation, promoting the generation of myeloid immunosuppressive cells (132). Biological function of the circulated T-EVs and their horizontal transfer of nucleic acids between distant cells have been reported in various studies. For example, Cossetti et al. demonstrated that human melanoma cells [transfected with enhanced green fluorescent protein (EGFP) plasmid] when injected into mice, released exosomes carrying EGFP RNA, that was found not only in the circulation but also in spermatozoa (133). Furthermore, EVs have also been reported to have the ability to shuttle viruses, such as HIV and EBV (124, 133–135). These data, therefore, suggest that EVs may potentially participate in the dynamic regulation of whole body functions. Since T-EVs exhibit specific molecular patterns and are present in the circulation, saliva, urine, and other biological fluids, they are considered as a new diagnostic non-invasive tool, whose reliability has been demonstrated by diverse clinical studies reported in the literature (136, 137).

Cancer stem cells, also known as “tumor-initiating cells,” are a subpopulation of cancer cells that have the ability to self-renew and give rise to new tumors and metastasis. At present, CSCs were identified in many solid tumors, such as breast, renal, ovary, brain, pancreatic, prostate, colon, melanomas, and hepatocellular cancers (138, 139). Several studies indicate that CSCs release EVs that may contribute to tumor initiation and progression by stimulation of cell proliferation, invasion, angiogenesis, and metastasis formation, as well as by the promotion of tumor immune escape (29, 140–143). In our laboratory, we have observed that EVs derived from renal CSCs impaired dendritic cell maturation as well as T cell immune response through HLA-G, a known suppressor of immune cell function and an effector of cancer immune escape (140). Moreover, these EVs promoted angiogenesis and lung metastases. Possible effectors of the above observed effects by EVs can be attributed to miRNAs carried by EVs, which are implicated in angiogenesis, tumor progression and metastases, such as miR-200c, miR-146, miR-92, miR-301, miR-7g, and miR-130b (29).

Extracellular vesicles derived from breast CXCR4-positive CSCs were shown to promote proliferation, motility, and metastasis, generating an enhanced tumorigenic phenotype in tumor cells (144). These EVs were highly enriched in mRNA of genes related with stem cell differentiation and development, such as NANOG, NEUROD1, HTR7, KISS1R, and HOXC6. Enrichment of these mRNA was detected also within EVs from plasma of breast cancer patients with poor prognosis, suggesting that CSCs-derived EVs could enhance cancer development by transporting RNA (144). Alessandro et al. (117) observed that CD90-positive CSCs from hepatocellular carcinoma released EVs, enriched with lncRNA H19, which upregulated VEGF in endothelial cells, stimulated angiogenesis, and promoted the adhesion of cancer cells to the endothelial cell monolayer. Furthermore, EVs from different cancer cell types were shown to contain different miRNA patterns. For instance, Sanchez et al. (145) demonstrated that EVs from bulk prostate cancer cell line and from prostate CSCs contained 19 differentially expressed miRNAs and showed collaborative biological effect in tumorigenic niche formation. Moreover, breast cancer cell lines released EVs were identified to be rich in mir-130a, which contributes to tumorigenesis of cancer by regulating TGB-β/Smad signaling (146), mir-106b that promotes breast cancer invasion and metastasis by targeting BRMS1 and RB (147), miR-210 that promotes angiogenesis and metastasis *in vivo* (148), and several others miRNAs (149).

As single miRNA can regulate multiple targets in different cells or tissues, single miRNA could act as a tumor suppressor in one context and as an oncogene in another. Let-7, miR-15b, miR-122, and miR-100 are examples of miRNAs that were detected in plasma of cancer patients and exhibited dual role in cancer development.

## Mesenchymal Stem Cells Could Regulate Tumor Growth via EVs

Tumor initiation and metastasis require formation of a favorable niche, which is a specific microenvironment that promotes tumor cell viability, proliferation, invasion, and involves several other types of cells, including MSCs. MSCs may enhance or suppress tumor progression and metastasis depending on doses and time of administration as well as on tumor model and stage (150). Their effects are considered to be dependent on paracrine mechanisms (150, 151) and EVs have been implicated as mediators of MSC actions (152).

Similar to MSCs, EVs derived from them also exhibit a controversial influence on the development of tumors. Several studies have shown that MSC-EVs promote tumor growth through different mechanisms, including miRNAs transfer. For example, Zhu et al. (153) showed that MSC-EVs enhanced VEGF expression in tumor cells by activating extracellular signal-regulated kinase1/2 (ERK1/2) pathway. Other research groups showed that MSC-EVs significantly increased survival of tumor cells and support tumor growth *in vivo* in part by mechanism involving miR-21 and miR-34a (6).

On the other hand, several studies have also demonstrated the inhibitory role of MSC-EVs on tumor growth. For instance, it has been described, that MSC-EVs markedly downregulated the expression of VEGF in tumor cells, inhibiting tumor angiogenesis, by a mechanism involving miR-16, which was abundant in MSC-EVs and predicted to target VEGF (74). In our laboratory, it was found that EVs derived from human bone marrow MSCs inhibited both *in vitro* and *in vivo* growth of HepG2, Kaposi, and Skov-3 cells (19). MSC-EVs induced a block in cell cycle progression in G0–G1 phase in all cell lines. Moreover, they induced apoptosis in HepG2 and Kaposi cells and necrosis in Skov-3 cells. The biological effect was explained by a differential regulation of genes involved in the control of cell cycle inducing arrest of proliferation, therefore causing cell death by apoptosis or necrosis. Similar inhibition of cell cycle progression with arrest in the G1 phase was reported for MSCs as well (154–156). This anti-proliferative effect of MSCs was related to soluble factors since the cell-to-cell contact was not required (157).

EVs derived from human liver stem cells (HLSCs) also exhibited an anti-tumor effect due to the transfer of anti-tumor miRNAs (miR-223, miR-24, miR-31, miR-125b, and miR-451). EVs obtained from Dicer knock-down HLSCs showed a significant reduction of anti-tumor activity both *in vitro* and *in vivo*. Furthermore, the inhibition of miR-451 and miR-31 reduced the observed pro-apoptotic activity of HLSC-EVs. When injecting HLSC-EVs or miR-31 or miR-451 mimics *in vivo* (intra-tumor), a regression in the tumor was observed (22). In another study, it has been shown that MSC-EVs carried miR-23b, which decreases proliferation and invasion of breast cancer cells through the inhibition of MARCKS promoter of cell motility and cycling (158). Notably, this miRNA was detected in T-EVs as well together with other anti-tumor miRNAs, such as miR-145, miR-143, miR-223, and miR-224. It has, therefore, been suggested that tumor cells possibly dismiss these miRNAs through EVs to achieve more metastatic properties (159).

This controversial data on the effect of MSC-EVs suggest that MSCs could release EVs that could have functions that are complex, diverse, and even opposite depending on the environment they are in. Even the same molecular pathways may respond in a different manner when stimulated by EVs. For instance, VEGF expression in tumor cells and consequent angiogenesis were shown either to be upregulated (153) or downregulated (74) after MSC-EV stimulation. The main difference between these studies showing anti-tumor or pro-tumor effect of MSC-EVs was the method of EV collection. When EVs were obtained from serum deprived MSCs they promoted tumor growth. In contrast, when EVs were collected from MSCs cultured with serum, they exhibited tumor-suppressive action. Indeed, Vallabhaneni et al. (6) showed that serum-deprivation may stress MSCs resulting in upregulation of miRNAs (miR-21 and miR-34) and lncRNA (lnc-Y1 and lnc-7SK) that are involved in cell survival and inhibition of apoptosis. These results suggest that cell stress stimulate production and secretion of molecules that may support tumor growth.

Another condition that changes the pro- or anti-tumoral characteristics of MSC-EVs is MSC priming by tumor cells. MSCs co-cultured with tumor cells or stimulated with T-EVs were shown to release EVs with tumorigenic properties. For example, in our laboratory, it was found that MSC stimulation with EVs from renal carcinoma CSCs leads to production of MSC-EVs that enhanced tumor cell migration and exhibited angiogenic properties (160). Another research group showed that MSC-EVs of patients with multiple myeloma-induced tumor growth *in vivo* and promoted dissemination of tumor cells to the bone morrow, whereas normal MSC-EVs exhibited an anti-tumor effect. Possible mechanism of this finding was the decreased content of tumor-suppressor miR-15a in EVs derived from multiple myeloma MSCs with respect to normal MSC-EVs (161).

As for the cells, the timing of EV administration may be critical (150). In fact, in the early phase of tumor growth, MSCs as well their EVs may facilitate the angiogenic shift of tumor favoring tumor initiation (162). By contrast, in established tumors, MSCs and MSC-EVs may promote apoptosis of endothelial cells and tumor regression (163). Therefore, MSC-EV influence on tumor growth may depend not only on type and stage of tumor but also on MSC culture conditions that may modify the cell secretome.

## Conclusion

Through various studies reported in the current literature, it is quite evident that EVs derived from both MSCs and tumor cells display common ncRNAs; however, the functions they exhibit may be diverse depending on the cellular environment. Since ncRNAs interact with numerous molecular partners, their function is complex and depends on the cellular context. The response to EVs not only depends on their ncRNA/protein content but also on the metabolic pathways activated in recipient cells as well as their specific function. The same EVs, for instance, may trigger opposite actions in normal and tumor cells (22). Pandolfi and co-workers (164) have suggested that there is a RNA language that uses miRNAs response elements in different transcripts as “letters.” These diverse sets of miRNAs may form diverse “words,” thus performing different molecular and cellular functions. This hypothesis may, therefore, explain the different functions carried out by the same miRNAs in different contexts on tumor development. Taking into account that EV transport is bidirectional and that tumor cells can change the content of MSC-EVs and *vice versa* (160, 161), it is possible that the individual ncRNA display different and even opposite functions *in vivo*.

## Author Contributions

All authors conceived and wrote the manuscript.

## Conflict of Interest Statement

The authors declare that the research was conducted in the absence of any commercial or financial relationships that could be construed as a potential conflict of interest.
